# A Volumetric Method for Quantifying Atherosclerosis in Mice by Using MicroCT: Comparison to *En Face*

**DOI:** 10.1371/journal.pone.0018800

**Published:** 2011-04-18

**Authors:** David J. Lloyd, Joan Helmering, Stephen A. Kaufman, James Turk, Matt Silva, Sergio Vasquez, David Weinstein, Brad Johnston, Clarence Hale, Murielle M. Véniant

**Affiliations:** 1 Department of Metabolic Disorders, Amgen Inc., Thousand Oaks, California, United States of America; 2 Department of Pathology, Amgen Inc., Thousand Oaks, California, United States of America; 3 Department of Research Imaging, Amgen Inc., Thousand Oaks, California, United States of America; 4 Numira Biosciences, Salt Lake City, Utah, United States of America; Universität Würzburg, Germany

## Abstract

Precise quantification of atherosclerotic plaque in preclinical models of atherosclerosis requires the volumetric assessment of the lesion(s) while maintaining *in situ* architecture. Here we use micro-computed tomography (microCT) to detect *ex vivo* aortic plaque established in three dyslipidemic mouse models of atherosclerosis. All three models lack the low-density lipoprotein receptor (Ldlr^−/−^), each differing in plaque severity, allowing the evaluation of different plaque volumes using microCT technology. From clearly identified lesions in the thoracic aorta from each model, we were able to determine plaque volume (0.04–3.1 mm^3^), intimal surface area (0.5–30 mm^2^), and maximum plaque (intimal-medial) thickness (0.1–0.7 mm). Further, quantification of aortic volume allowed calculation of vessel occlusion by the plaque. To validate microCT for future preclinical studies, we compared microCT data to intimal surface area (by using *en face* methodology). Both plaque surface area and plaque volume were in excellent correlation between microCT assessment and *en face* surface area (r^2^ = 0.99, p<0.0001 and r^2^ = 0.95, p<0.0001, respectively). MicroCT also identified internal characteristics of the lipid core and fibrous cap, which were confirmed pathologically as Stary type III-V lesions. These data validate the use of microCT technology to provide a more exact empirical measure of *ex vivo* plaque volume throughout the entire intact aorta *in situ* for the quantification of atherosclerosis in preclinical models.

## Introduction

Ischemic heart disease resulting from coronary atherosclerosis is the leading cause of human mortality worldwide [1], [2]. Atherosclerosis is characterized by deposition of cholesterol ester in the arteries that may progressively occlude the lumen and become prone to rupture and thrombosis. Therapies aimed at reducing the risk factors for developing atherosclerosis and/or directly reducing the size of the plaque are being actively pursued, creating the need for more robust methods to determine plaque alterations in preclinical models of atherosclerosis.

To date, different methods have been used to assess the extent of atherosclerosis in mice, or quantitatively compare lesion formation in various strains or treatment groups of mice. One method requires the sequential sectioning of the heart and aortic root [3] and subsequent histopathologic analysis to score and measure lesions in a 300 micron area at the level of the aortic sinus. Another method employs Sudan IV staining to determine the extent of atherosclerosis affecting the intimal surface throughout the entire aorta [4]. This method is commonly referred to as the *en face* technique and has been widely used as it yields morphometric data [5]. The technique consists of dissecting the aorta from the heart to the iliac bifurcation, opening it longitudinally to expose the luminal side, and staining it with Sudan IV to reveal lipid-laden plaques to measure lesional surface areas. These two methods provide qualitatively distinct estimates of lesion area in orthogonal axes. Another method uses high-resolution magnetic resonance imaging (MRI) to detect and quantify the plaque volume incorporating all dimensions assessed by the previous two methods in the intact aorta and to detect the lipid-rich necrotic core without requiring histopathology [6]. Despite the advancements in assessing aortic plaque, the need remains for the generation of very high-resolution three-dimensional plaque models, along with determining the degree of occlusion.

Currently, only the aortic root (Paigen) method is still routinely used for assessing the volume of an atherosclerotic lesion in preclinical models [3]. Though widely used, it is quite labor intensive and generates only a calculated estimate of aortic lesions. The estimate is often made on the basis of surface areas that are determined from only 10 sections (typically 10 µm) at fixed distances from the aortic sinus. The surface areas in turn are used to estimate the volume between the dropped sections. This approach provides only a limited volumetric assessment in the aortic root, and does not include the rest of the aorta or the adjoining arteries. We focused our work on validating the microcomputed tomography (microCT) method by comparing it with the *en face* method. This was done because the *en face* method was previously validated by comparing it with the aortic root Paigen method [4], [7] and because *en face* assesses the entire aorta and not just 10 transversal sections of the aortic roots.

While traditional histopathology analysis remains the gold-standard technique for the assessment of *ex vivo* tissues, these methods are labor intensive, time consuming, and often rely on subjective, qualitative measures. For that reason, there is increasing use of medical imaging technologies, primarily micro-MRI and CT, as alternatives or supplements to histopathology. When coupled with appropriate contrast agents, these methods can highlight tissue with high resolution and three-dimensional visualization. These methods are non-destructive, enabling scanning of the entire fixed tissue and allowing for subsequent histopathology. Finally, a key advantage of the imaging methodologies is the 3D visualization and analysis options wherein image segmentation and analysis algorithms quantify visualized pathologies into statistically measurable parameters. Because of the high contrast for bone, microCT has traditionally been used for the analysis of bone to examine changes in density and structure in response to treatments [8], [9], [10], [11]). However, recent method and contrast development have allowed improved imaging of soft tissue [12], [13], [14] and vessels [15], [16], [17], facilitating the assessment of disease states.

Along these lines, the feasibility of microCT has been used to assess atherosclerosis in 8 coronary arteries from human autopsy specimens [18] with excellent correlation to histological findings. In this report we demonstrate the use of microCT in 3 mouse models with different levels of atherosclerotic lesions all associated with deficiency of the Ldlr. Recently, the same technology was applied to lesion detection in *ApoE* ^−/−^ mice [19]. Here we extend these data and formally validate microCT for use in preclinical atherosclerosis by comparison with the conventional *en face* method and found the results from the two methods were in excellent correlation.

## Materials and Methods

### Ethics Statement

All animal studies were approved by the Amgen Inc. IACUC under protocol number 2006-00010.

### Animals

Four male Ldlr-3KO mice (*Ldlr* ^−/−^ *Apob* ^100/100^ *Lep* ^ob/ob^) and 4 male Ldlr-2KO mice (*Ldlr* ^−/−^ *Apob* ^100/100^ *Lep* ^ob/+^) were bred at Charles River Laboratories (San Diego, CA). The generation of the mice has been discussed previously [20]. Mice were fed standard chow (8640; Harlan Teklad; Indianapolis, IN) for the duration of the study. Five *Ldlr* ^−/−^ (Ldlr-1KO) mice were obtained from Jackson Laboratories (Bar Harbor, ME) were fed standard chow until they were 12 weeks of age, at which point they were fed an atherogenic (TD.02028; Harlan Teklad) diet for 16 weeks.

### Plasma analysis

Four-hour fasted plasma was collected in Ldlr-2KO and -3KO mice (20-21 weeks of age). Mice were bled from the retro-orbital sinus, and blood was collected into EDTA plasma tubes. Plasma lipids were measured using the Olympus AU400e Chemistry Analyzer (Olympus America, Inc; Center Valley, PA).

### Perfusion and sample preparation

A whole-body cardiac perfusion was performed three to four days after plasma collection. The mice were anesthetized with a 50 mg/kg intraperitoneal injection of Nembutal. Anesthetized mice were perfused with phosphate-buffered saline (PBS) followed by fixative (4% paraformaldehyde, 5% sucrose, 20 mM EDTA, pH 7.4) in the absence of a nitric oxide donor. The perfusion fixation was done using an *in vivo* perfusion system (AutoMate Scientific Inc., San Francisco, CA) following the instruction manual. We calculated that 53 mmHg was the estimated pressure we used to perfuse each mouse. Whole mouse carcasses were not used for imaging due to the size constraints of scanning necessary for sufficient resolution; instead carcasses were trimmed and the excised heart and aorta specimens intact with spine, ribs and kidneys were shipped in fixative to Numira Biosciences (Salt Lake City, UT) for microCT imaging and quantitation. Specimens were allowed to fix by gentle agitation in 10% neutral-buffered formalin for an additional 4–5 days at room temperature.

After complete fixation the samples were immersed in a 5% Phosphotungstic Acid solution for 48 (±2) hours. The samples were rinsed with 1x PBS both before and after reagent exposure. The described specimen preparation, staining, and scanning process is a patent-pending method of Numira Biosciences Inc (www.numirabio.com). Numira performs this method as a service to its clients. The contrast reagent was only used during the soaking of the carcass and not injected during perfusion of the mouse. Full immersion of the sample was necessary for uptake of the agent into the aorta and plaque to allow them to be distinguished from each other.

### MicroCT imaging and data processing

MicroCT Imaging: A high-resolution, volumetric microCT scanner (µCT40; ScanCo Medical, Zurich, CH) was used to scan the tissue with the following parameters: 10 µm isometric voxel resolution, 200 ms per view, 2000 views in a 360 degree rotation, 55 kVP for tube voltage and averaging the five frames into one. The scanners utilized in this study are calibrated weekly as recommended by the manufacturer. The calibration block is supplied by the manufacturer and contains a metal rod of known volume. The instrument scans the block and calculates the volume of the rod and the calculated volume must be within 2.0% of the actual volume. Initially only 1 cm of the descending aorta was scanned (for Ldlr-2KO and -3KO mice), in later experiments (Ldlr-1KO AD mice) the aorta were scanned to the iliac bifurcation. The microCT data was reconstructed and files were converted to DICOM format for further processing.

Image Processing: Seg3D, a segmentation software package from the Scientific Computing and Imaging Institute (SCI, University of Utah, Salt Lake City, UT) was used to semi-automatically label plaques within the aortic arch. No filtering was used during the instrument reconstruction process. The aorta was first labeled based on prior knowledge of its cylindrical shape and the grayscale values of its wall. To differentiate the different areas, plaque, lumen and aorta, the basic grayscale criteria differences were used, as each entity has a unique value that can be distinguished from the others. The software was then used to label the appropriate areas.

The software applications Teem (http://teem.sourceforge.net/) and SCIRun (SCI) were then used to calculate the plaque volumes and surface areas, and to create the intimal-medial thickness-map movies. Presence of plaques was verified by an external radiology consultant (MicroRad, LLC; Salt Lake City, UT).

Volume Measurements: As part of the segmentation process, Seg3D reported the number of voxels associated with the aorta and plaque. The voxel count for each region of interest was converted into cubic millimeters by multiplying by the volume per voxel element.

Total Surface Area Measurements: To create smooth surface representations of the regions of interest, the boundary faces of the segmented regions were smoothed using Taubin's algorithm [21]. The surface area for each region was then calculated by summing the areas of all of the triangles associated with each boundary.

Attached Plaque Surface Area: We define the attached portion of the plaque as the region of the surface that abuts the aorta surface (in contrast to that portion of the surface that faces the interior of the aorta). The “Attached Plaque Surface Area” measurement was also used when computing the “Percent Coverage” (percentage of aorta wall that is covered by plaque), as seen in the “5-Panel Percent Occlusion Map” movies. Since the attached plaque surface is relatively smooth and represents just one side of the three-dimensional plaque surface, it was anticipated that the Intimal (attached) Plaque Surface Area would correspond well with the plaque surface area measured with the en face method.

Intimal-Medial Thickness Map Generation: For each point on the plaque surface, we computed the minimum distance to the opposite side of the plaque (ie, vessel-side to lumen-side and vice versa). The plaque was pseudo-colored to indicate the plaque thickness at each point by using the color map shown on the left corner of the thickness map images.

### *En face* analysis

After Numira Biosciences completed the CT analysis, aortas were soaked for one week in 4% paraformaldehyde/7.5% sucrose fixative to remove the CT contrast agent as well as to make the aorta more flexible for dissection. Aortas were excised from the carcass, separated from the heart, flat-mounted on wax boards, and stained with Sudan IV using previously published methods [20]. After staining, aortas were trimmed to yield the aortic arch and a 1 cm section of the descending aorta of Ldlr-2KO and -3KO mice, or the entire aorta of Ldlr-1KO AD mice. The segments were individually positioned on a glass plate and mounted under a glass coverslip using PBS, eliminating all folds and air bubbles. Images of the aortas were captured with a Nikon DXM 1200 digital camera using a Nikon SMZ-U dissecting microscope (Nikon USA; Melville, NY) and the ACT1 controller software. Each image was analyzed with Metamorph Imaging System software v6.1 (Molecular Devices Corp., Sunnyvale, CA) using RGB thresholding to define lesion areas. Atherosclerotic lesions were quantified by a trained operator blinded to mouse genotype.

### Histological analysis

After *en face* imaging, the Sudan IV stained aortic whole mount samples from mice were returned to fixative. Later, by using both the *en face* and microCT images as a guide to assist in trimming, the aorta lengths were transected at specific points, yielding segments several millimeters in length, and were processed in paraffin using standard histological methods. Once infiltrated, the specimens were embedded perpendicularly in blocks with the trim edge down, providing selected cross-sectional profiles across the aorta. Sequential sections were stained with H&E, Verhoeff-Van Gieson, and picrosirius red. Lesions were graded according to Stary type [22], [23].

### MicroCT reproducibility assessment

The reproducibility of microCT measurements was assessed by four independent operators who were instructed to analyze the microCT images for an aorta and to derive lesion surface areas and volumes. We calculated the coefficient of variation (CV) to assess reproducibility.

### Statistical analysis

Data analysis was performed using GraphPad Prism 5 (GraphPad Software, Inc.). Linear regression and Spearman's Rank Correlation coefficient calculations were performed to compare the lesional data generated by the microCT and *en face* methodologies. Data in Table 1 comparing Ldlr-2KO and -3KO mice were analyzed using a Student's T-Test; data comparing all three strains were analysed by using a One-Way ANOVA and a Tukey post hoc test.

**Table 1:** Aortic and lesion dimensional characteristics of Ldlr-1KO mice fed atherogenic diet (AD) and Ldlr-2KO and -3KO mice as determined by microCT and *en face*.

			Ldlr-1KO-AD^a^		Mean +/− SEM		Ldlr-2KO^b^		Mean +/− SEM		Ldlr-3KO^b^		Mean +/− SEM			
Aorta No.	4	5	7	17	18		6941	6928	6930	6962		6927	6943	6944^c^	6945	
microCT																
Total Aorta Volume (mm^3^)^d^	11.7	10.2	11.1	10.6	11.2	11.1±0.3	7.2	6.2	7.42	8.2	7.3±0.4	8.5	8.3	23.6	12.3	13.2±3.6 *
Total Plaque Volume (mm^3^)^d^	2.5	3.1	3	2.5	2.3	2.7±0.2	0.1	0	0	0.1	0.1±0.0	0.5	0.4	0.7	0.7	0.6±0.1 **
Plaque volume (mm^3^)/Aorta volume (mm^3^)^e^	0.21	0.30	0.27	0.24	0.21	0.25±0.02	0.01	0.00	0.00	0.01	0.01±0.00 ∧∧∧	0.06	0.05	0.03	0.06	0.05±0.01 ∧∧∧ &
Total Aorta Surface Area (mm^2^)^d^	63.1	50.9	53.5	52.2	52.4	54.4±2.2	33.2	28.1	29.7	36.8	32±1.9	34.3	35.3	70.7	45.4	46.4±8.5
Total Attached Plaque Surface Area (mm^2^)^d^	22.2	21.7	30	19.2	19.6	22.54±2.0	0.5	0	0	1	0.4±0.2	4.2	5.3	7.9	8.3	6.4±1.0 **
Plaque area (mm^2^)/Aorta area (mm^2^)^e^	0.35	0.43	0.56	0.37	0.37	0.42±0.04	0.02	0	0	0.03	0.01±0.01 ∧∧∧	0.12	0.15	0.11	0.18	0.14±0.01 ∧∧∧ &
Maximum Plaque Thickness (mm)^d^	0.4	0.7	0.3	0.5	0.4	0.5±0.1	0.1	0	0	0.1	0.1±0.0	0.5	0.2	0.2	0.2	0.3±0.1 *
Average % Occlusion^d^	24.6	34.1	35.7	26.1	23.4	28.8±2.5	−	−	−	−	−	−	−	−	−	−
*En Face*																
Total Plaque Surface Area (mm^2^)^d^	14.9	13.5	16.8	12.4	11.6	13.8±0.9	0.4	0.1	0.2	0.7	0.4±0.1	2.6	3.7	8.4	5.5	5.1±1.3 *

a Aortas scanned from aortic valve to illiac bifurcation.

b Aortas scanned from aortic valve to 1 cm down descending aorta.

c Aorta with aneurysm.

d Only data from Ldlr-2KO and -3KO were analysed using a T-Test, as they were generated similarly (1 cm of descending aorta).

e Data from all three strains (Ldlr-1KO-AD, -2KO and -3KO) were analyzed using ANOVA, as these readouts of plaque were standardized to aorta dimension.

* P<0.05;

** P<0.01 *vs.* Ldlr-2KO aortas using a T-Test.

∧∧∧P<0.0001 *vs.* Ldlr-1KO AD aortas,

& P<0.05 *vs.* Ldlr-2KO aortas using ANOVA and Tukey post hoc test.

## Results

### Detection of atherosclerotic plaque in Ldlr-2KO and -3KO mice

**Figure 1:**
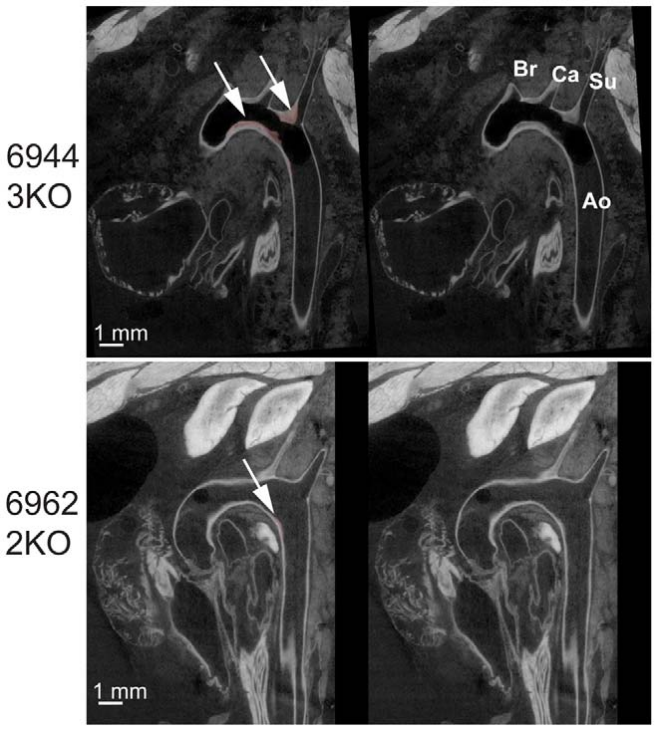
Coronal microCT image of the aortic arch. A microCT section of 10 µm is shown for an Ldlr-3KO aorta (top 2 images) and an Ldlr-2KO aorta (bottom two images). The descending aorta (Ao) and brachiocephalic (Br), left common carotid (Ca) and left subclavian (Su) arteries are clearly seen. Aortic lesion is evident within the luminal space of the aorta and can be distinguished from the aortic wall by the difference in gray scale values of the CT data between the aortic wall and the lesion and is demarcated in pink after segmentation (arrows; images on left).

**Figure 2:**
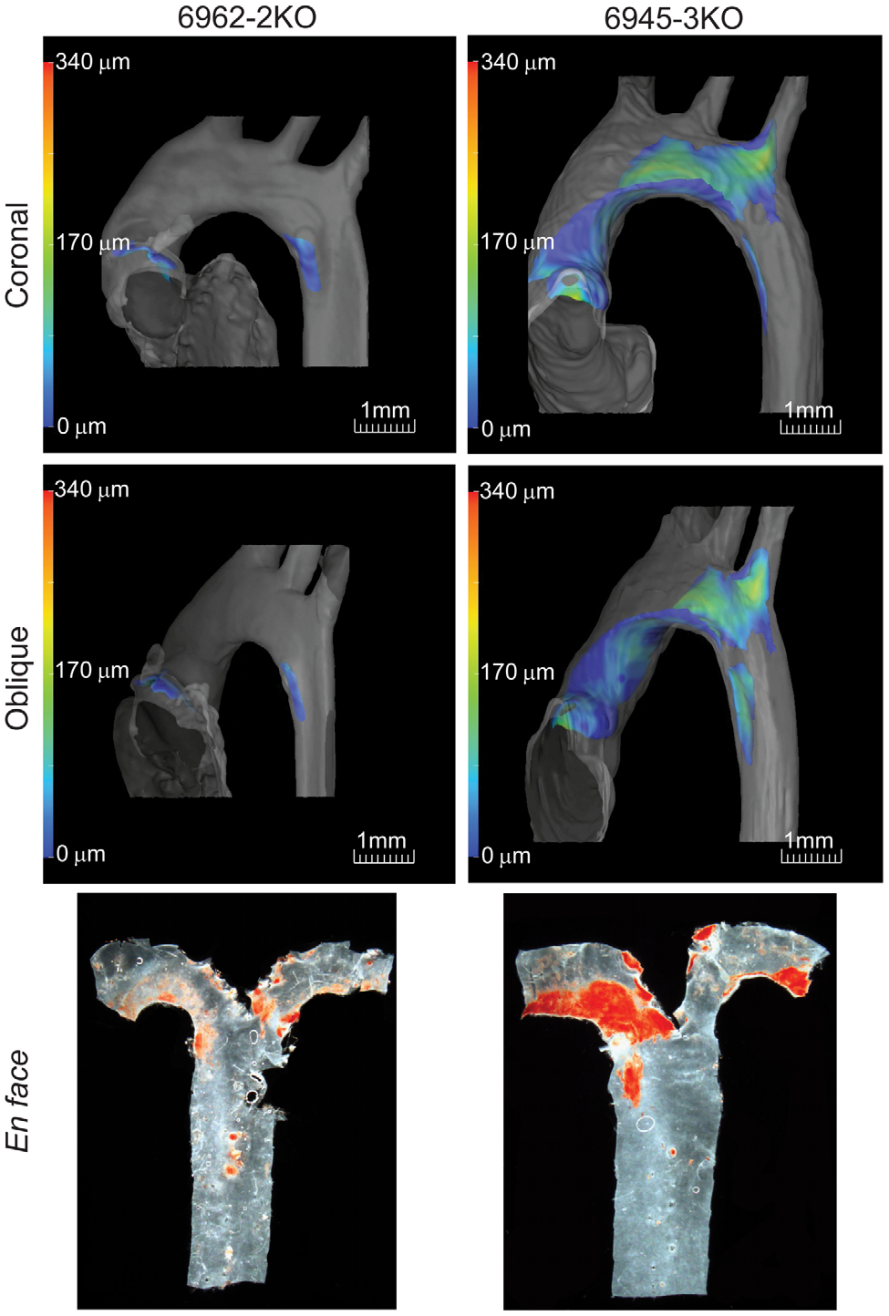
Three-dimensional renderings of atherosclerotic plaque. The microCT images are rendered to generate a 3D representation of the aortic arch and attached plaque. A rotating movie (Movie S1) allows the viewing of the plaque from any aspect. Coronal and oblique aspects obtained from the movies are shown for both Ldlr-2KO (left images) and -3KO mice (right images). The distance of the surface of the plaque from the aorta luminal wall is indicated using a color spectrum scale (0–340 µm) shown on the left-hand side of each image. Both coronal and oblique sections illustrate differences in the amount of atherosclerosis detected by microCT between the two strains. The same aortas were excised from the carcass and assessed by *en face* methods (see Materials and Methods) to allow comparison of the aortic arch plaque by using the two techniques.

Ldlr-2KO and -3KO mice were used to evaluate the feasibility of identifying plaque in the aortas using microCT since they exhibit different amounts of plaque. As expected, the Ldlr-3KO mice were obese, extremely hypercholesterolemic (including elevated HDL-cholesterol) and hypertriglyceridemic compared with Ldlr-2KO mice (Table S1, [20]). The Ldlr-2KO mice were also hypercholesterolemic compared with typical levels in wildtype mice (Table S1, [24]). Two-dimensional microCT images were generated and used to visualize the aorta and resident plaque from the Ldlr-2KO and -3KO mice. Figure 1 shows a representative 10 µm microCT coronal section of the aorta and arteries and clearly defines artery wall, lumen and plaque. The aorta and lesions were segmented from the microCT dataset and a 3D rendering of the microCT data was generated. A rotational loop sequence of these frames was then generated and composited into a movie (Movie S1). Single images from the movies were captured to compare the aortas from the two mouse strains side-by-side (Figure 2). Since linear measurements were generated throughout the aortas and lesions, plaque thickness was modeled. It is important to note that microCT detection of the plaque area was confirmed with the use of the conventional *en face* staining technique in the same aortas. Figure 2 (lower panels, *en face*) confirms the gross 2D shape of the lesions found in each aorta.

Each aorta from Ldlr-2KO and -3KO mice was measured using microCT analysis (Table 1) and *en face* methods (Figure S1). We also calculated plaque area normalized to total aorta area, for comparison between the mouse strains. As expected, smaller lesion sizes were found in the Ldlr-2KO mice than in Ldlr-3KO mice. In two Ldlr-2KO mice no lesions were detected by microCT, whereas small areas of Sudan IV-positive staining were evident from *en face* analyses (Figure S1, aortas 6928 and 6930). Interestingly, a severe aneurysm was observed in the aorta from an Ldlr-3KO mouse (aorta 6944; Figures S1 and S2). The vessel volume was approximately double that of the other Ldlr-3KO mice; however, the plaque volume was not similarly increased.

We found that both readouts of lesion surface and volume were highly reproducible; plaque surface area – SD = 0.59, CV = 5.25%, plaque volume – SD = 0.014, CV = 2.98%.

We evaluated whether the lesion measurements between Ldlr-2KO and -3KO aortas generated with microCT showed similar fold differences with *en face* (Figure S3) and found a 14.6-fold difference in total plaque surface area between Ldlr-2KO and Ldlr-3KO using *en face* and a 17.5-fold difference using microCT (Figure S3A), demonstrating comparable fold differences between the two techniques. We also calculated an 11.7-fold difference in plaque volume between the Ldlr-2KO and Ldlr-3KO aortas (Figure S3B).

### Detection of atherosclerotic plaque in a standard preclinical model of atherogenesis

**Figure 3:**
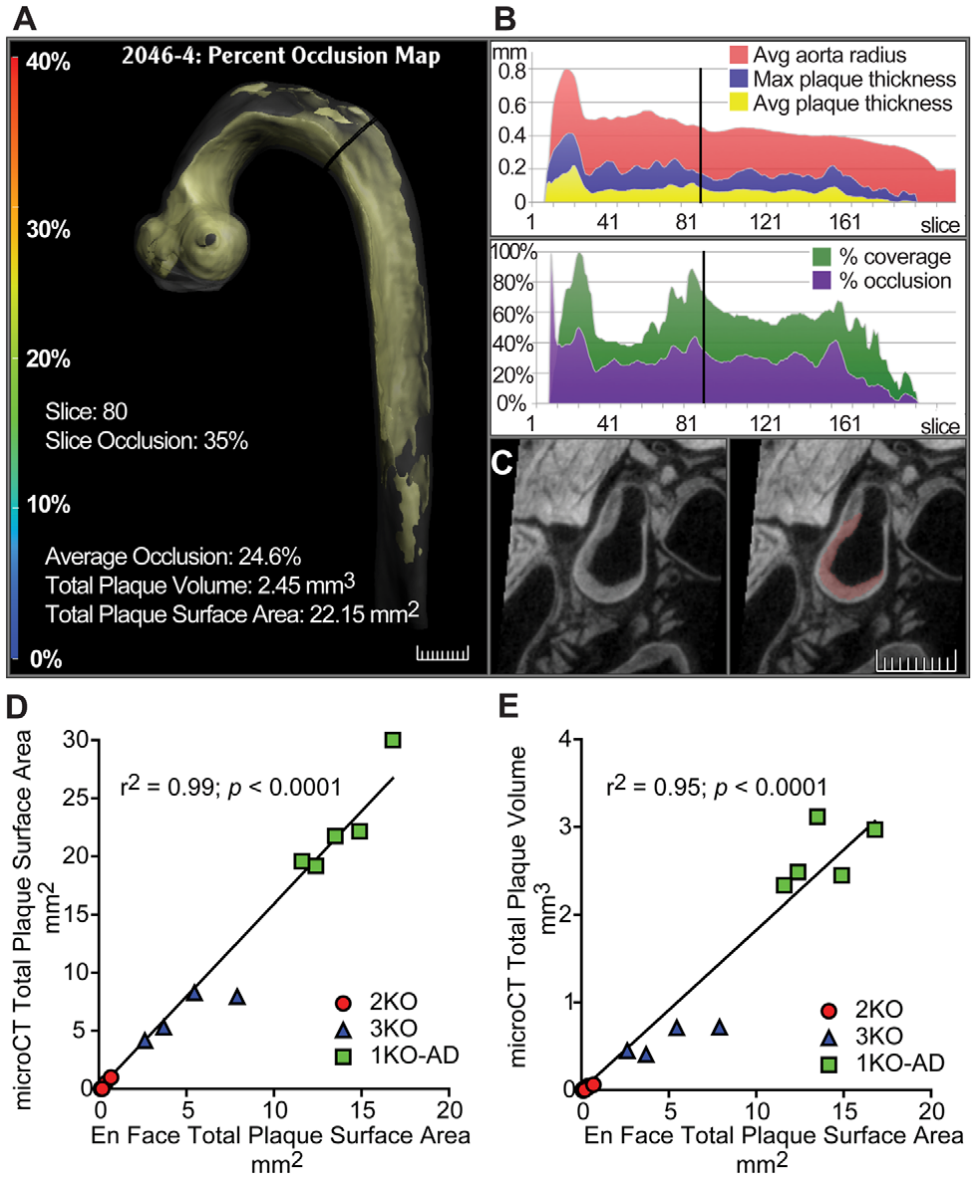
Plaque Detection by microCT in Ldlr-1KO atherogenic diet-fed mice; correlation of *en face* with microCT. A single frame of a 5-panel movie (Movie S2) of an Ldlr-1KO fed an atherogenic diet. (A) Aortic arch and descending aorta (gray) showing location of the plaque (yellow). Cross section of aorta is show by the black line to indicate the section of interest in subsequent panels. (B) Both upper and lower graphs represent sections along the aorta (proximal to distal) along the x–axis and plaque dimensions along the y–axis. Upper graph shows average aorta radius (red), maximum plaque thickness (blue) and average plaque thickness (yellow) at each section is shown continuously along the aorta. Lower graphs show percent plaque coverage (green) and percent occlusion of the plaque within the aorta. The vertical line in both graphs indicates position in relation to section in panel A. (C) MicroCT image of the section indicated in panels A and B shows a cross section of the aorta with accumulation of plaque. The plaque which is detected by the imaging has been demarcated in pink (right image). (D, E) Correlation of lesional readouts using microCT and *en face*. *En face* plaque surface area data derived from aortas of Ldlr-1KO-fed atherogenic diet (AD) (green squares), -2KO (red circles), and -3KO (blue triangles) mice were compared with plaque surface area (D) or plaque volume (E) derived by microCT methodology. Linear regression and correlation coefficients were calculated for each data set comparison.

To evaluate microCT for its utility in preclinical studies, we quantitatively analyzed atherosclerotic plaques in 5 Ldlr single knockout mice fed an atherogenic diet (Ldlr-1KO-AD) (a common model of atherogenesis [25], [26], [27], [28]. In these mice plasma cholesterol levels were extremely elevated (3137 +/− 63 mg/dL). Similarly, the number of aortic plaques from the Ldlr-1KO-AD was higher than those from Ldlr-2KO and -3KO mice (Table 1 and Figure 3). In these analyses, 5-panel movies incorporating many images and readouts were created (See Methods and Movie S2). Figure 3A–C shows a single frame from a 5-panel movie. Extensive atherosclerotic lesions in the Ldlr-1KO-AD throughout the aortic tree and descending aorta are clear, along with other dimensions of the aorta, lesions and lumen (% occlusion). *En face* surface areas were generated for each aorta (Table 1 and Figure S4) and were used to determine correlation between the two methods.

### Correlation of microCT with *en face*

To assess the correlation of the surface areas generated by microCT and by the *en face* techniques, data from each aorta for all three mouse strains were graphed (Figure 3D). The two methods are in near-perfect correlation (r^2^ = 0.99; *p*<0.0001). To determine whether the microCT volumetric readout is an appropriate predictor of plaque quantity, and to confirm its correlation with *en face* surface areas, we compared the two sets of data from the three strains (Figure 3E). We found almost perfect correlation (r^2^ = 0.95, *p*<0.0001) demonstrating that microCT plaque volume are in excellent agreement with the typical *en face* measurements.

### Histological analyses after microCT

**Figure 4:**
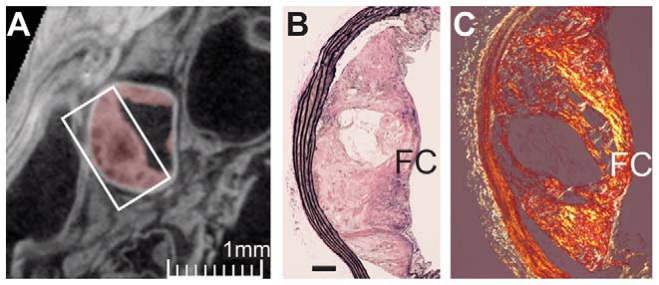
Histological examination of aortas pre-selected by microCT and Sudanophilia. (A) MicroCT cross-section of an aorta from an Ldlr-1KO mouse revealed a lesion (demarcated in pink) occluding 69% of the aorta with variable internal density. (B) Verhoeff-Van Gieson stained section of the raised lesion outlined by the white rectangle in 4A contained a Type V fibroatheroma (Figure 4B) with a core of extracellular lipid rimmed by foam cells (corresponding to the dark center observed by microCT) and overlain by a fibrous cap (FC) that is better defined by birefringence on polarization of the same lesion stained with picrosirius red (C). Bar in C represents 100 microns.

To determine feasibility of histologic evaluation following microCT analysis, sections of aortas from Ldlr-1KO-AD mice preselected by microCT and en face Sudanophilia were assessed. These sections contained plaques consistent with Stary types I-V; a thin endothelial layer, covering a fibrous cap was evident, as were foam cells and lipid-rich necrotic core. Raised lesions that were heterogeneous in density by microCT (Figure 4A) contained Type V fibroatheroma with a fibrous cap overlying core of extracellular lipid rimmed by foam cells (Figures 4B, C). The analysis demonstrates preservation of pathological architecture following microCT scanning, and histological focusing based on microCT images.

## Discussion

Imaging technologies have the advantage of both *in vivo* and *ex vivo* applications, with the latter offering increased resolution, sensitivity, and ease as the operator is not constrained by complications of working with a live animal. For these studies, the *ex vivo* validation was deemed necessary as proof-of-concept prior to executing method development for *in vivo* imaging studies. MicroCT imaging offers high-resolution (6–36 µm for the device used in this study) and isotropic voxels for true 3D quantification and visualization. Several examples of microCT-based analysis of bone diseases have demonstrated the power of this technique [8], [9], [10], [11]. More recent work has demonstrated that improved imaging methods and contrast reagents can enable microCT to be used for soft tissue [17], [18], [14], [29] and vessel imaging [15], [16], [17].

The microCT methodology presented clearly detected aortic lesions in all three Ldlr -deficient models, providing data that can be processed to generate movies to view the relevant features from any orientation. In the first example, we produced a rotating movie around a ventral/dorsal axis (Movie S1), allowing viewing of the aorta and plaque from all perspectives. In the second example we produced a multipanel movie that travels through the aorta, displaying relevant dimensional statistics with reference to the original microCT images (Movie S2). Importantly, we determined the precise amount of lesional volume and calculated luminal occlusion by the lesions based on volumetric data. The attached surface area and volume of the lesions determined by microCT were highly reproducible between operators (CV = 5.3% and 3% respectively) and correlated highly (*p*<0.0001) to *en face* surface areas. In addition to quantifying the dimensions of the plaque, we found that microCT can also yield information about the interior of the lesion, notably the necrotic area confirmed by histologic analysis, without resorting to those methods that may that may also alter the morphology and therefore affect volumetric measurements.

The use of microCT to detect lesions in mice offers numerous advantages over traditional techniques. 1) From the perspective of the researcher, hands-on time is possibly reduced; the aortas need not be precisely removed from the animal and only minimal organ trimming is necessary. Although staining with a contrast agent takes 2 days, the aortas once positioned within the CT scanner, can be scanned for overnight data collection, and data processing and image/3D rendering is mostly performed *in silico*. The current method also reduces artifact or operator error as the collection of data is performed in an unbiased manner by software that has been provided guidance to determine the area of plaques by trained personnel. 2) Accurate volumetric data can be collected which reflect the precise volume of the lesion and aorta, unlike the aortic root in which estimations of plaque volume are used between dropped sections. Furthermore, microCT generates volumetric data of the aorta that is mostly still within its normal environment, potentially retaining all of its *in vivo* characteristics (with the exception of blood volume/displacement). In contrast, the aorta may be physically distorted by handling, shrunken by fixation/embedding with *en face* and aortic root sectioning.

The aneurysm detected in one of our Ldlr-3KO mice is a good example of the benefits of *in situ* 3D (Figure S2) imaging over the flattened *en face* preparation, where the architecture of this lesion was significantly distorted (aorta 6944; Figure S1). The *in situ* microCT scanning also allows the viewing and analysis of surrounding tissues and organs, for example the carotid arteries or perivascular adipose. 3) Anatomical planes can be chosen after processing, as opposed to the careful selection of one anatomical plane before traditional histologic analysis, or cutting of the tissue before *en face* analysis. One can choose the desired orientation of a particular histological projection by simple digital re-orientation of a data set. 4) The nondestructive *in situ* methodology leaves the aorta intact for future analyses, including histology to confirm potential necrotic areas and immunohistochemistry. Further, the precise area of the aorta can be selected for follow up based on reference to the microCT data. 5) Lastly internal composition of the lesions, specifically necrotic areas, can be identified. Presumably microCT will also be of use in those models of advanced atherosclerosis that exhibit lesions harboring areas of calcification.

Despite the obvious quantitative value of microCT imaging, there are significant hurdles to wide-spread use, including the cost and technical expertise required. Further, there are perhaps some limitations on the ability of microCT to detect the smallest of lesions. In two of the Ldlr-2KO mice we analyzed tiny lipid-positive areas that were detected and measured (both less than 0.2 mm^2^) by the *en face* method (Figure S1, Table 1) but that were not detected in these same samples by microCT. Further studies will be required to optimize the detection of lesions within this range.

The methodology described in this paper does not allow for *in vivo* microCT scanning due to the preparation and procedures needed to view the plaques. Here we describe a novel method for imaging plaques as a first step. The next step of the methodology will be to image animals *in vivo* to view the progression of the disease state. However, this will take development efforts to establish a procedure to view and accurately quantify the plaque.

In conclusion, this is the first study to validate the use of microCT to detect atherosclerosis in mice by comparing it with the established *en face* methodology. Additionally this procedure leaves the intact aorta available for follow-up histologic analyses. The microCT method will improve the precise volumetric evaluation of atherosclerotic plaques throughout the whole arterial tree in small animals because the preparative steps cause minimal morphological alterations. In addition, histology methods can be applied at the regions of interest after identification with micro-CT. These techniques can be applied together to further our understanding of pathologies in genetically modified models and to evaluate therapeutic efficacies in both prevention and regression models of atherosclerosis.

## Supporting Information

Figure S1 ***En face*** **assessment of atherosclerotic plaque.** Aortas from Ldlr-2KO (top images) and -3KO mice (bottom images) used for microCT analysis were dissected from the carcass and assessed by *en face* methods and Sudan IV staining to allow comparison of the aortic arch plaque using the 2 techniques. Lesional surface areas are reported in Table 1.
(DOC)

Figure S2 **Three-dimensional microCT rendering of aortic aneurysm.** A coronal aspect obtained from a rotating 3D movie (not shown) of aorta 6944 (Table 1 and Figures S1 and S4) displaying an aneurysm. The distance of the surface of the plaque from the aorta luminal wall is indicated using a color scale in micrometers shown on the left-hand side.
(DOC)

Figure S3 **Comparison of microCT to** ***en face*** **.** (A) Fold differences between the total intimal plaque surface area in Ldlr-2KO and -3KO aortas were calculated for each method. (B) The fold difference in total plaque volume determined by microCT was calculated between the two strains of mice. Region of the total aorta is described in Methods section.
(DOC)

Figure S4 ***En face*** **assessment of atherosclerotic plaque in Ldlr-1KO-AD mice.** Aortas from Ldlr-1KO fed atherogenic diet used for microCT analysis were dissected from the carcass and assessed by *en face* methods and Sudan IV staining to allow comparison of the aortic arch plaque using the 2 techniques. Lesional surface areas are reported in Table 1. Although the entire descending aorta was analyzed and reported, here we show only the aortic arch.
(DOC)

Table S1 **Body weight and plasma lipid characteristic of Ldlr-2KO and -3KO mice (N = 4).** Ldlr-3KO mice were obese, extremely hypercholesterolemic (including elevated HDL-cholesterol) and hypertriglyceridemic compared with Ldlr-2KO mice. The Ldlr-2KO mice were hypercholesterolemic compared with typical levels in wildtype mice.
(DOC)

Movie S1 **Intimal-medial plaque thickness movie of Ldlr-3KO aorta 6945 (** **Table 1** **):** Seg3D (Scientific Computing and Imaging Institute, University of Utah, Salt Lake City, UT) was used to create a label map associated with the regions of interest that assigned a specific value (1 for aorta, 2 for plaque and 0 for background). This label map was then used to produce a 3D rendering of the surface boundaries of the regions of interest: Aortic vessel in transparent gray and plaques in color spectrum (violet – thin plaque areas, to red – thick plaque areas). SCIRun (SCI Institute, Salt Lake City, UT) was used to generate the frames for the rotating 3D movies. The frames were then converted into QuickTime (Apple, Inc) movies for viewing.
(MOV)

Movie S2 **5-Panel Percent Occlusion Map Movie of Ldlr-1KO-AD aorta 4 (** **Table 1** **):** This movie ties together the three-dimensional aortic arch with the segmentation and quantitative metrics. The movie is split into five panels; each panel is described below: 3D Rendering Frame (left): we display the 3D aortic arch, with a perpendicular slice. The arch is rendered as two surfaces: the vessel is shown in a transparent gray; the plaque is shown in dark yellow. At the bottom of this frame, we report the slice number and slice percent occlusion as the movie progresses (and indicated by the black ring), as well as total specimen measurements for the average occlusion, total plaque volume, and total attached surface area. Linear Distance Measurements (top-right): for each slice along the aorta, we graph the average aorta radius, maximum plaque thickness, and the average plaque thickness. As the animation proceeds, a black time-bar indicates the current slice position. Percent Measurements (middle-right): for each slice along the curved centerline, we graph the percent coverage (percent of the vessel wall that has plaque attached) and percent occlusion (percent of the vessel cross-section that is occluded with plaque). As the animation proceeds, a black time-bar indicates the current slice position. Planar Slice (bottom-middle): slice of microCT volume that cuts through and is centered on the aorta, and which is oriented perpendicular to the centerline. Planar Slice with Segmentation Overlay (bottom-right): planar slice (same as above) with plaque segmentation overlaid in red. Total Percent Occlusion: The total percent occlusion for each specimen is taken as the average of the percent occlusion values calculated at each centerline-reformatted slice. We note that this formulation is different than the value that results from the ratio of the total plaque volume to the total vessel volume (which we do not report).
(MOV)
