# Supplementary figures and images for: A Volumetric Method for Quantifying Atherosclerosis in Mice by Using MicroCT: Comparison to En Face
Source: PLoS One. 2011 Apr 18;6(4):e18800. doi: 10.1371/journal.pone.0018800 (PMC3078927; doi:10.1371/journal.pone.0018800)

**Figure S1**


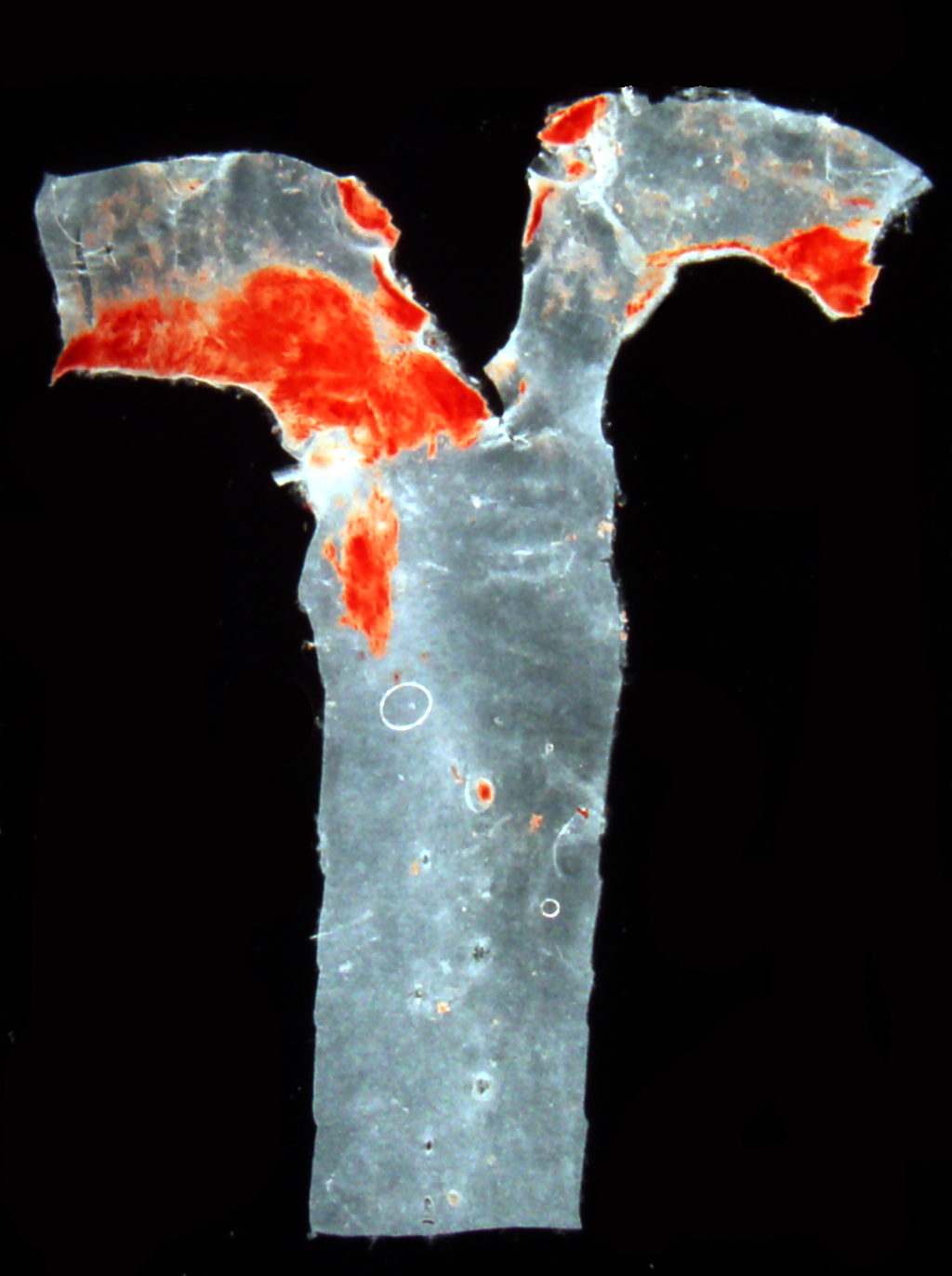


6945

**2 mm**


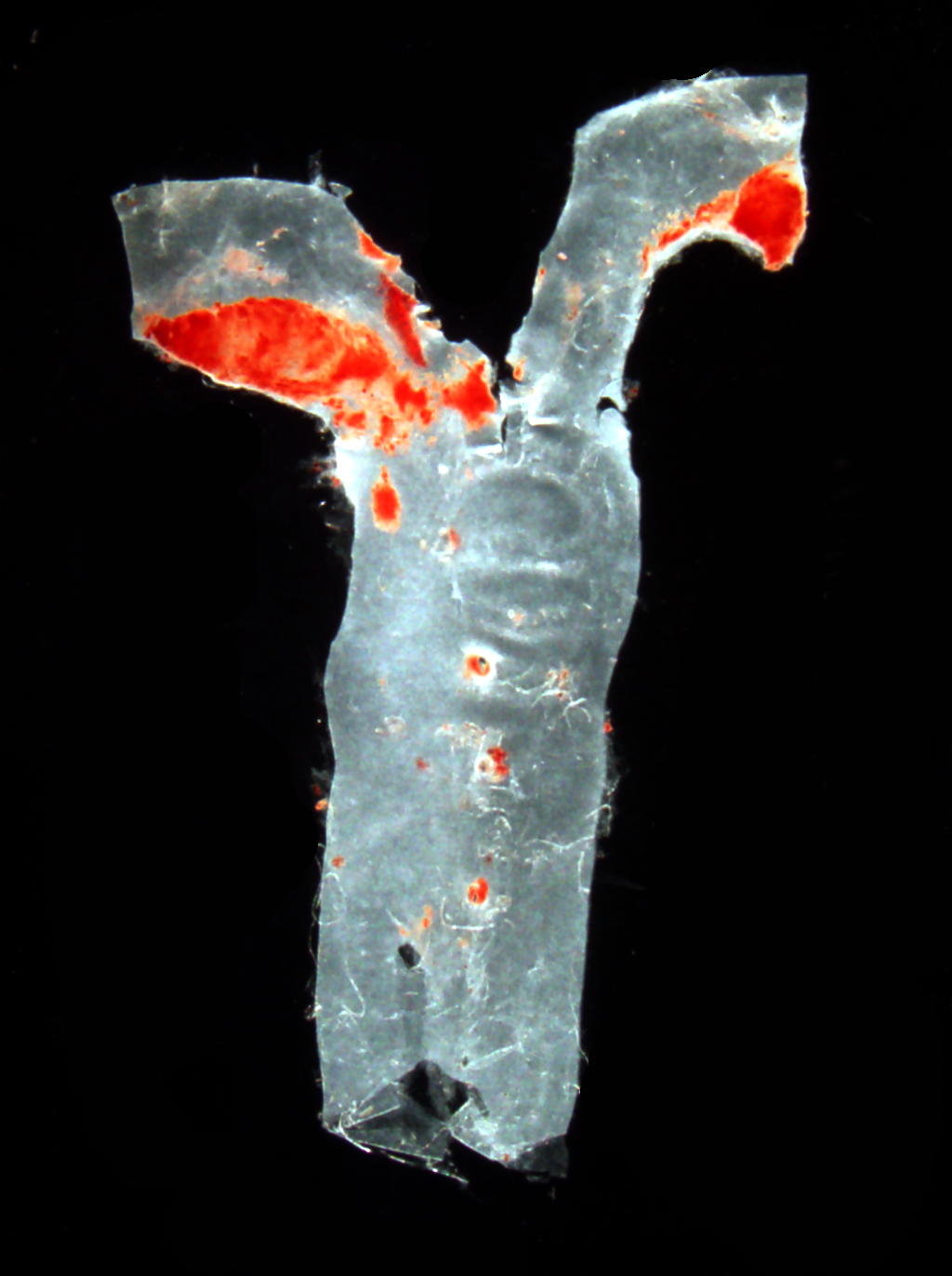


6927


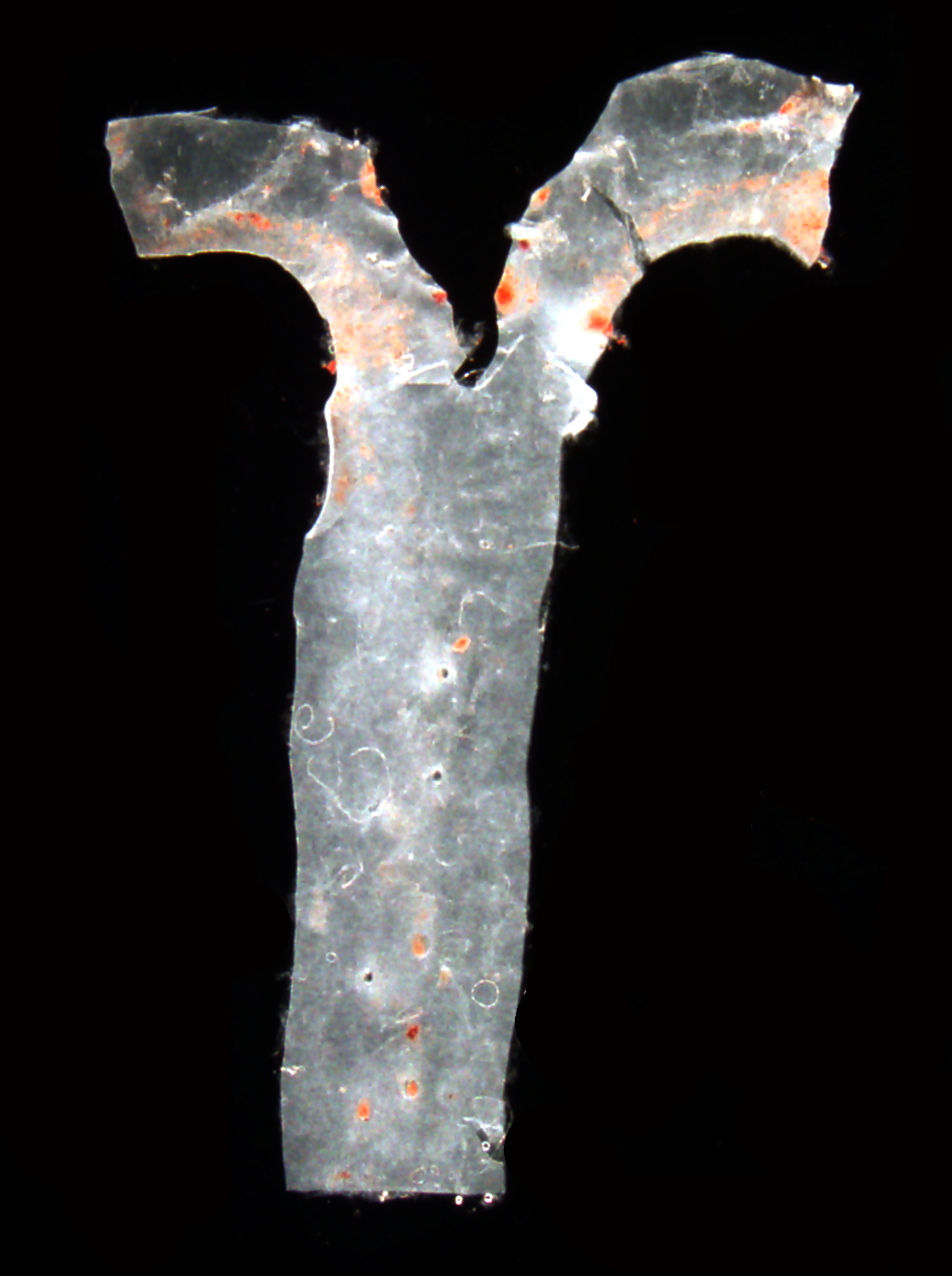


6928


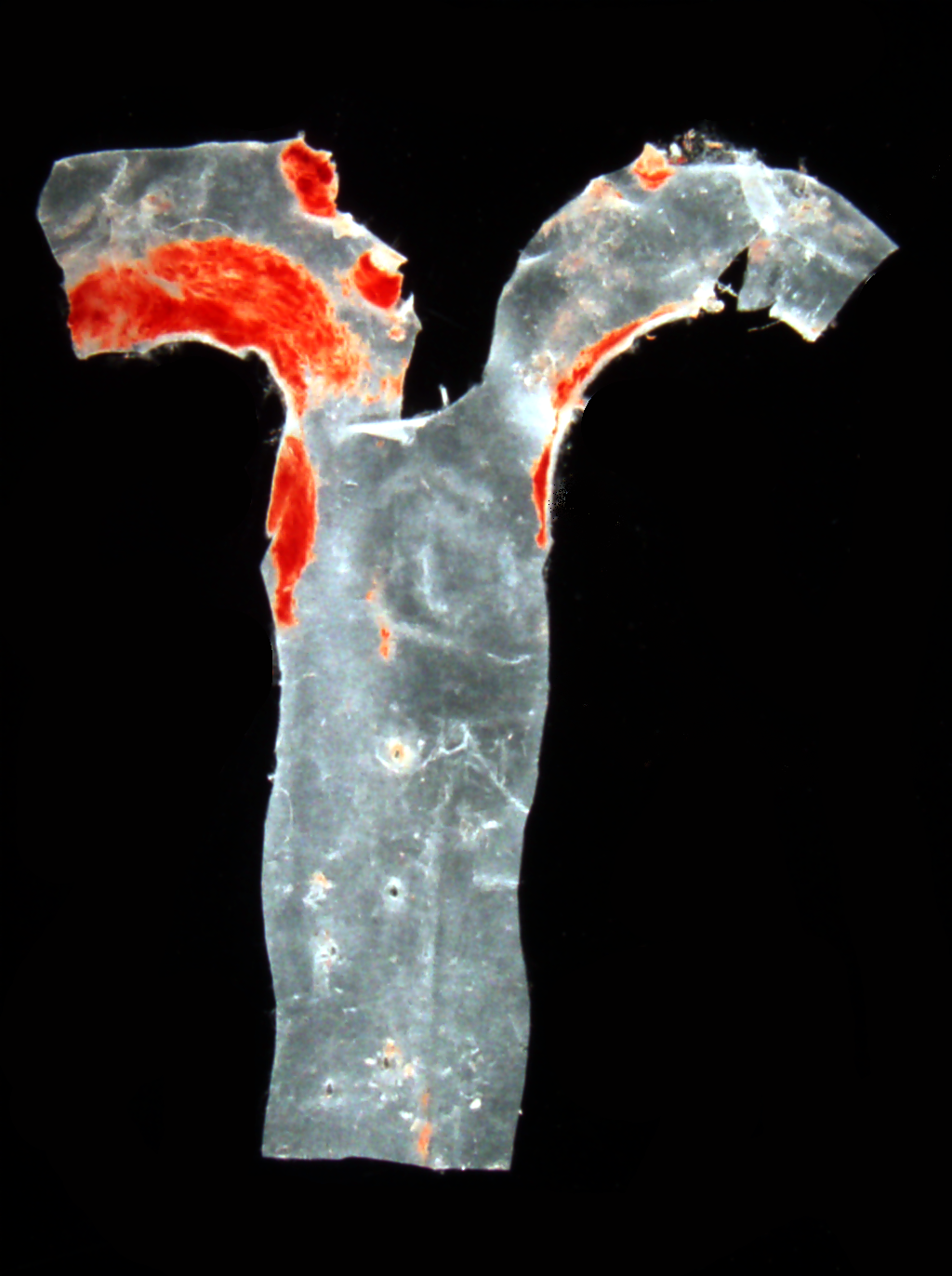


6943


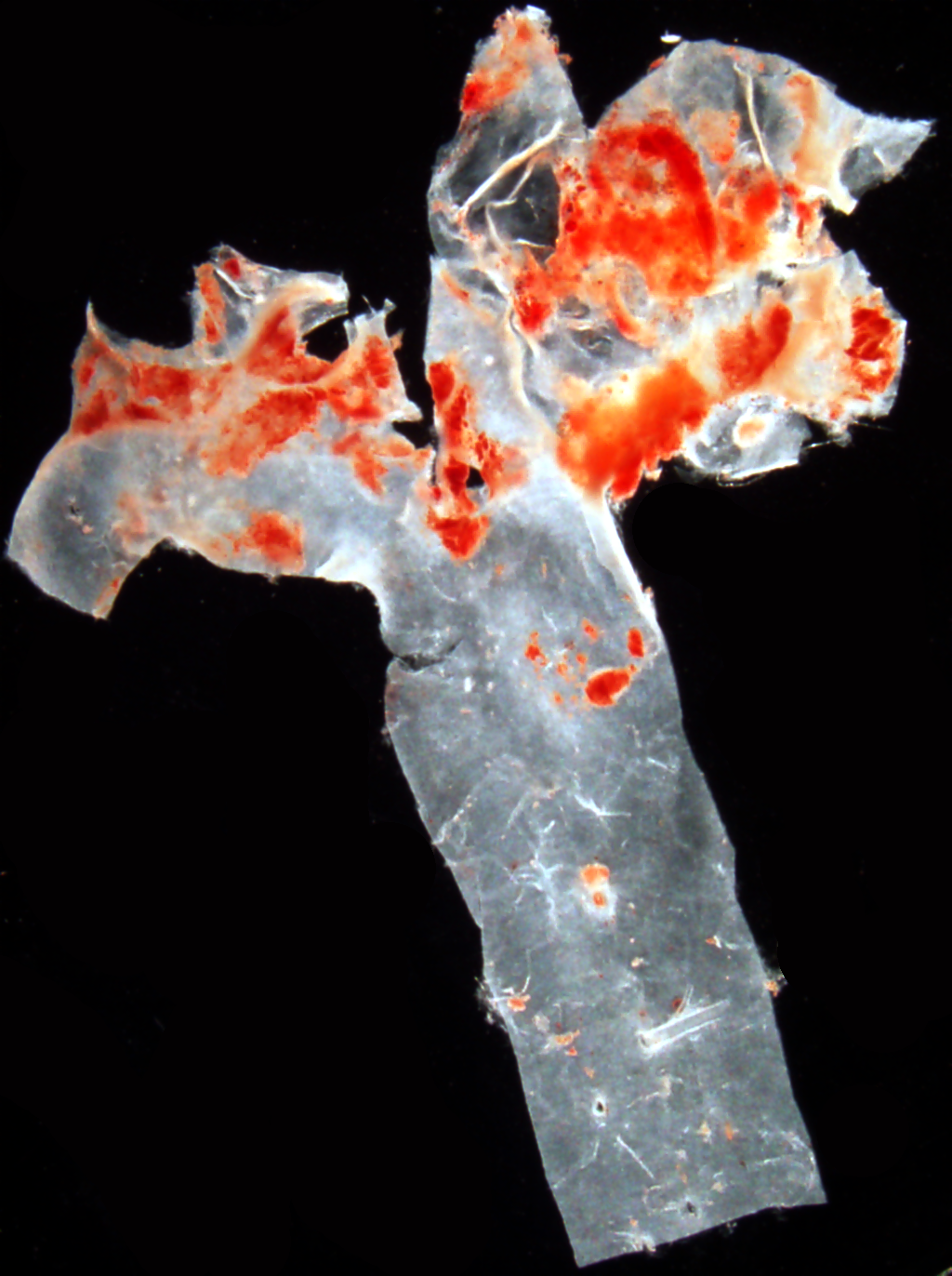


6944


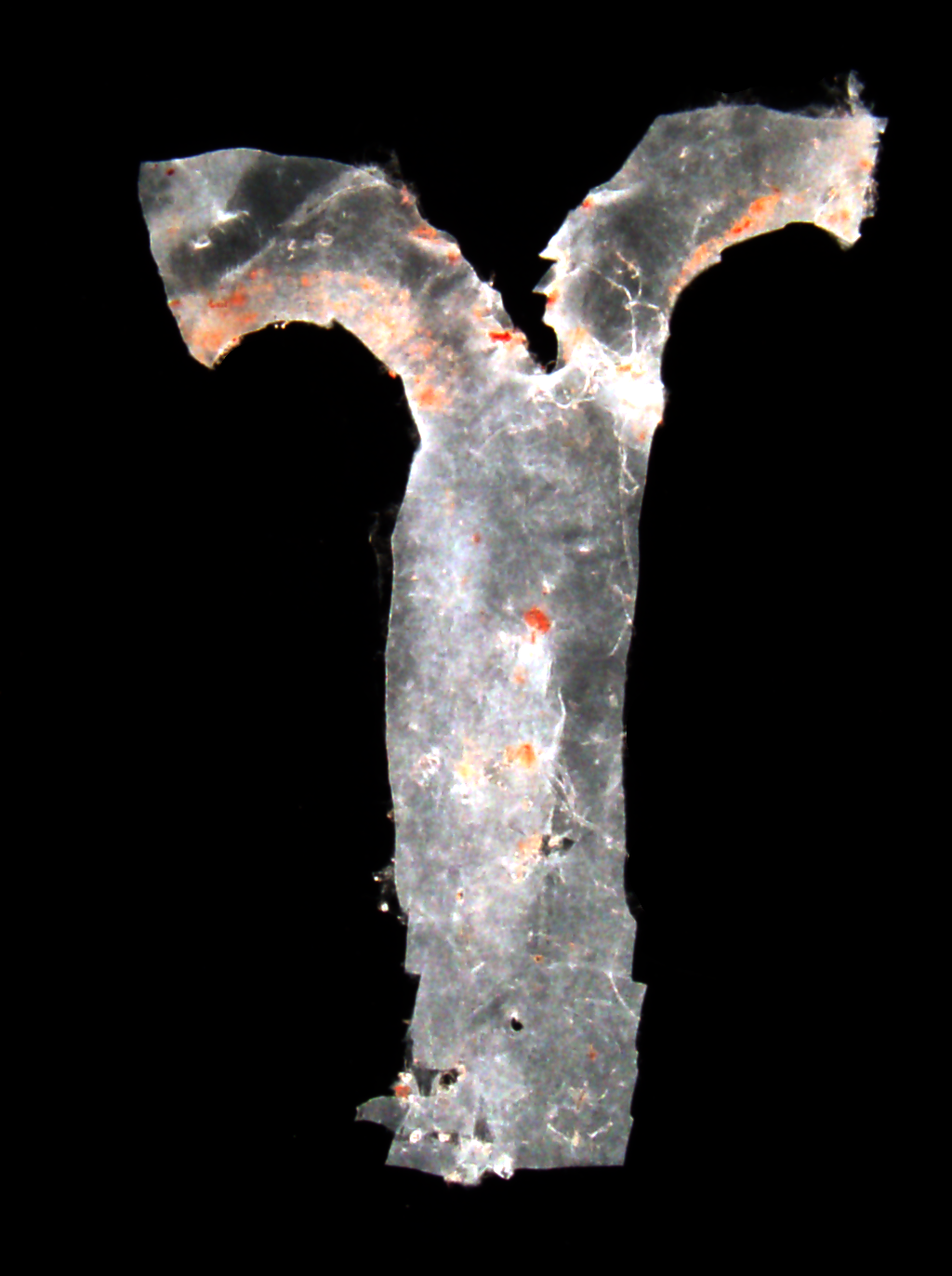


6930


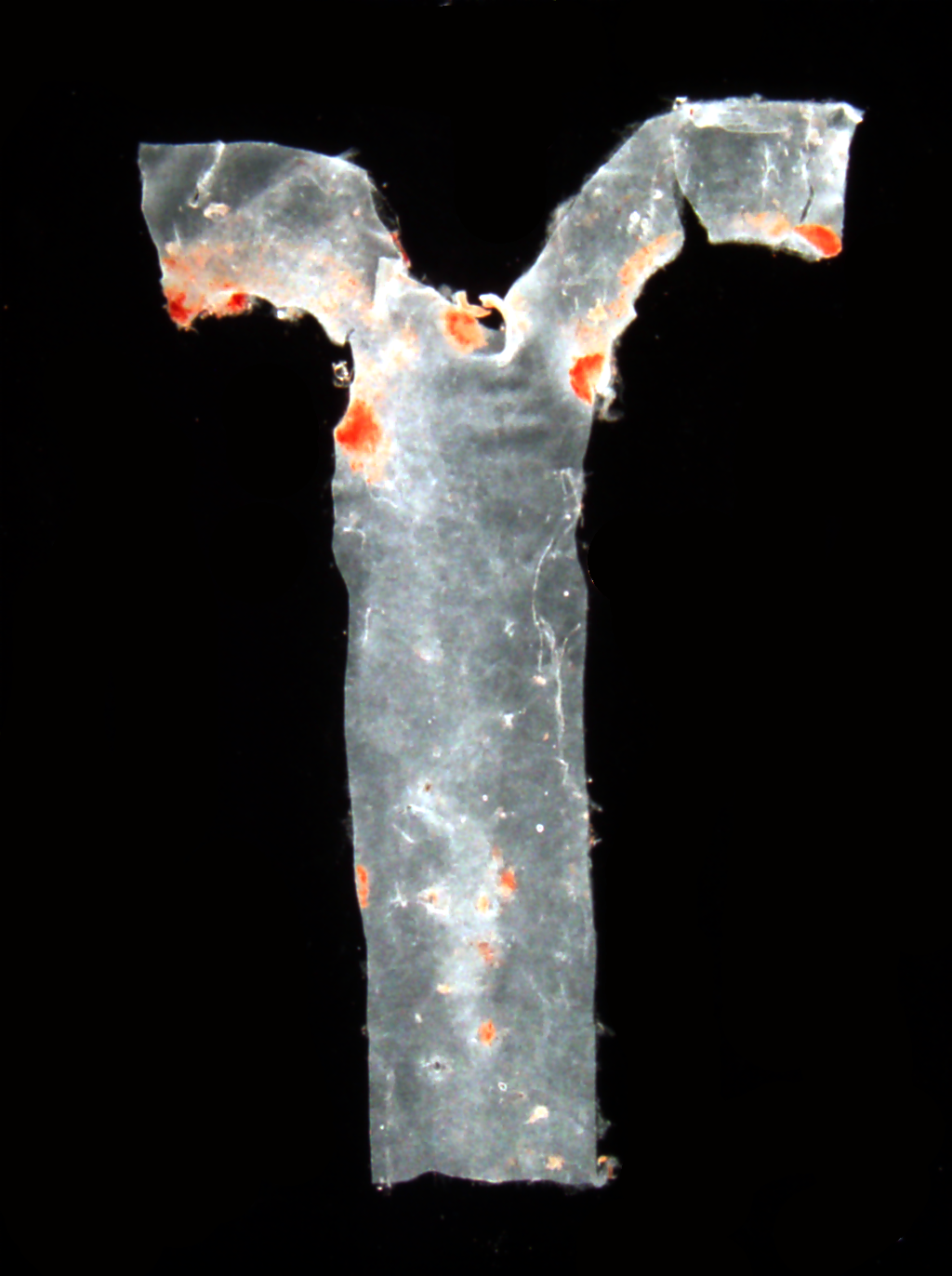


6941


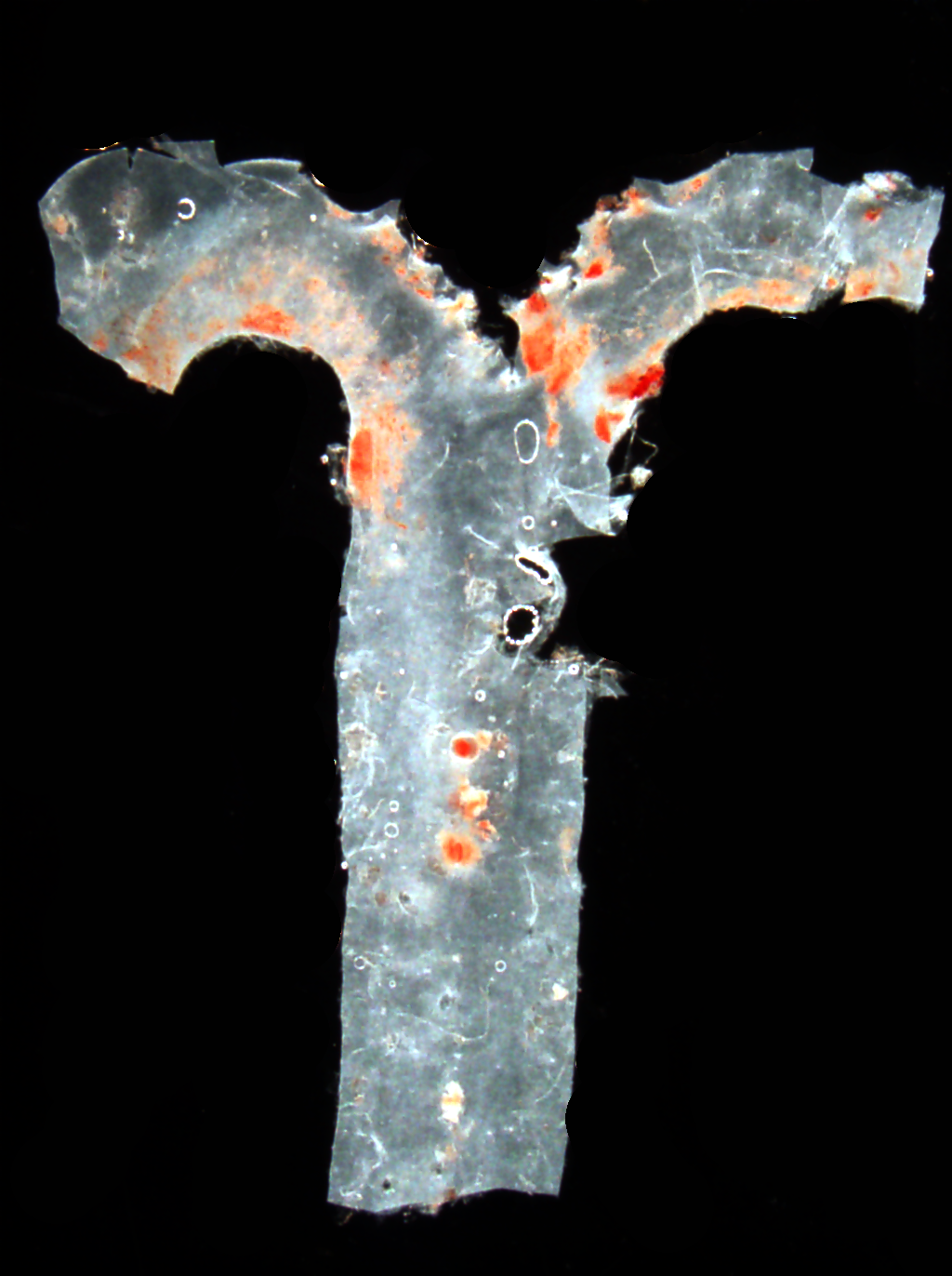


6962

**Ldlr-2KO**

**Ldlr-3KO**

Supplement: Figure S1 — En face assessment of atherosclerotic plaque. Aortas from Ldlr-2KO (top images) and -3KO mice (bottom images) used for microCT analysis were dissected from the carcass and assessed by en face methods and Sudan IV staining to allow comparison of the aortic arch plaque using the 2 techniques. Lesional surface areas are reported in Table 1. (DOC) [file pone.0018800.s001.doc]

**Figure S4**


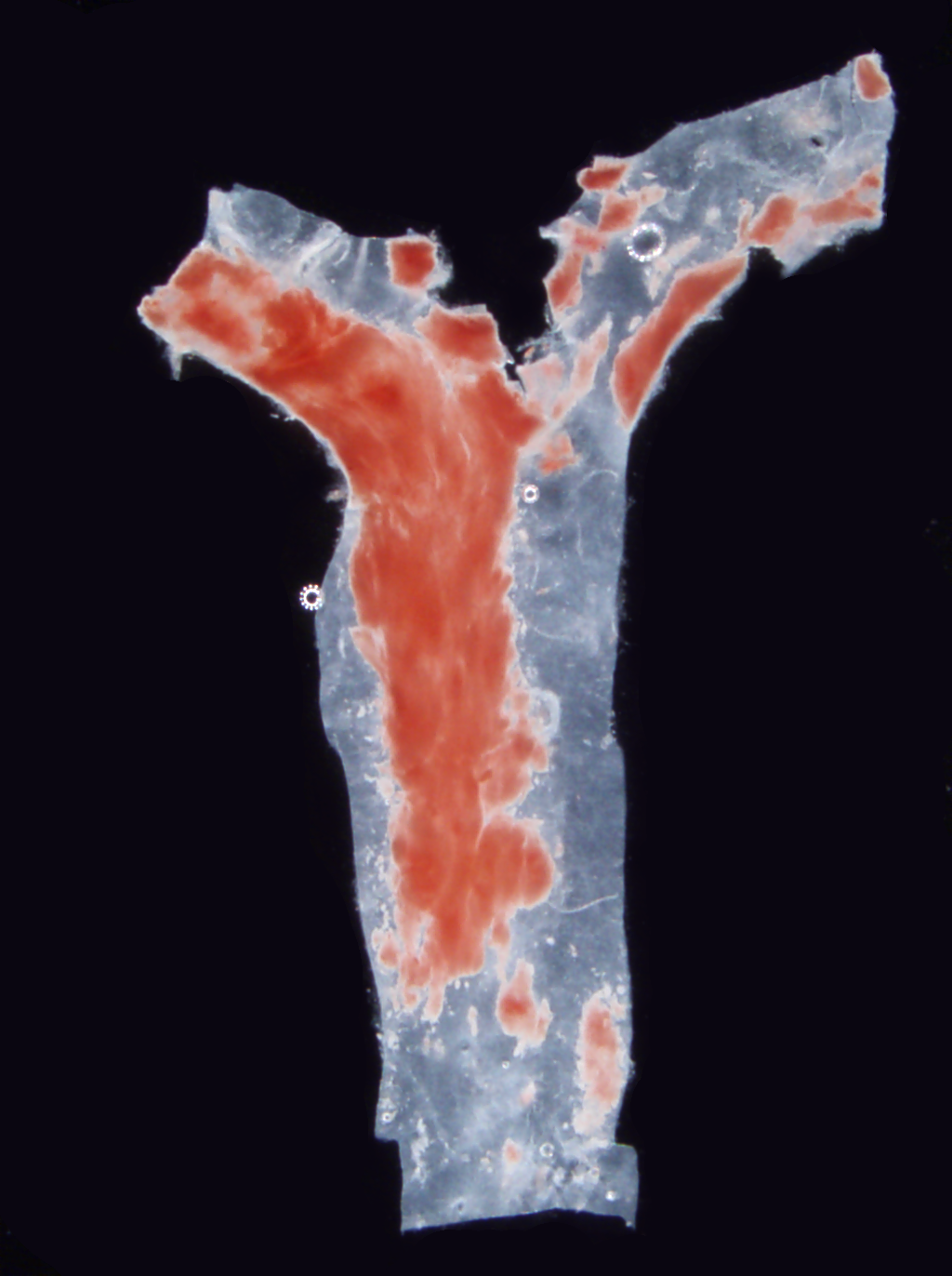


04


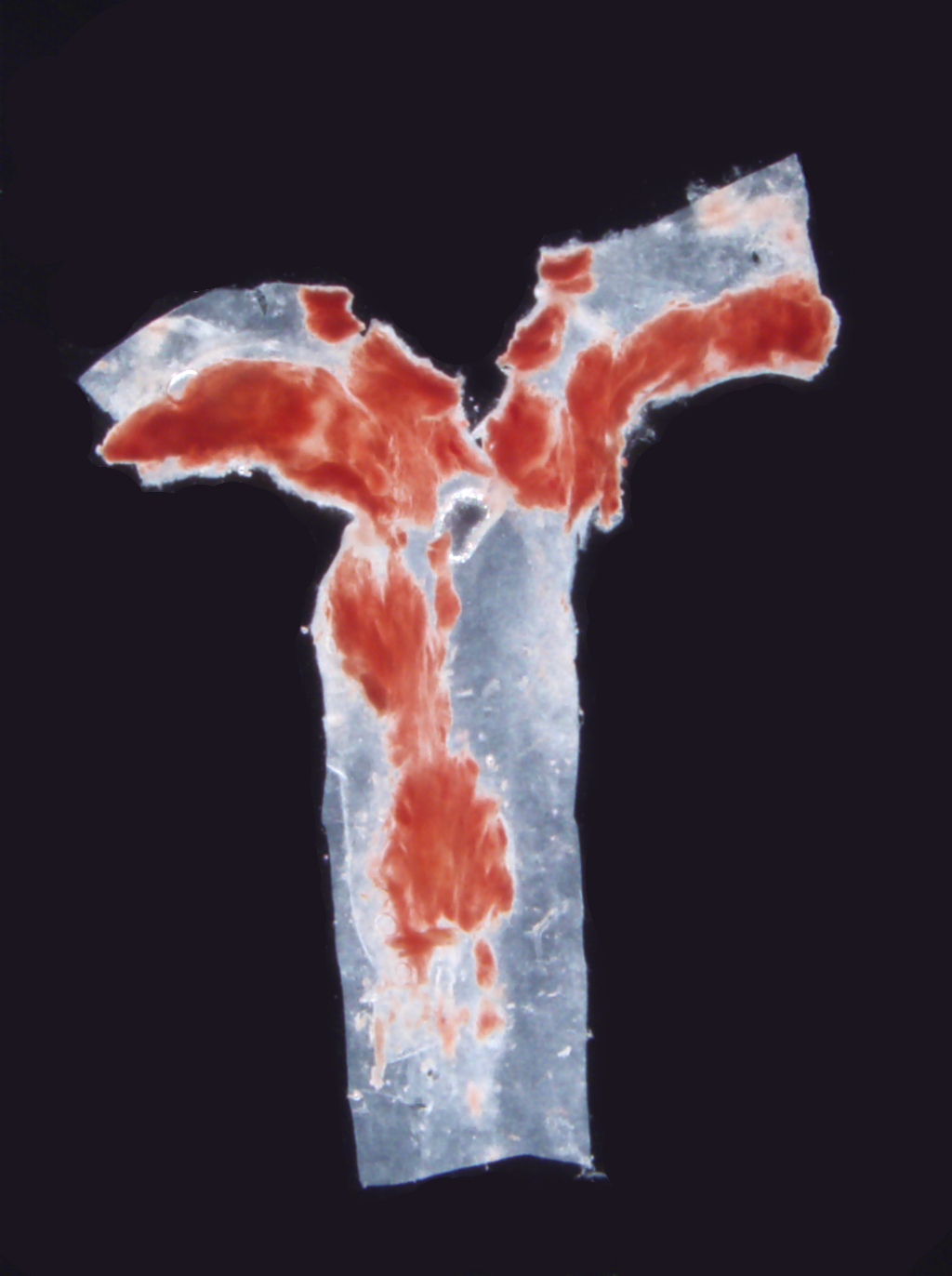


05


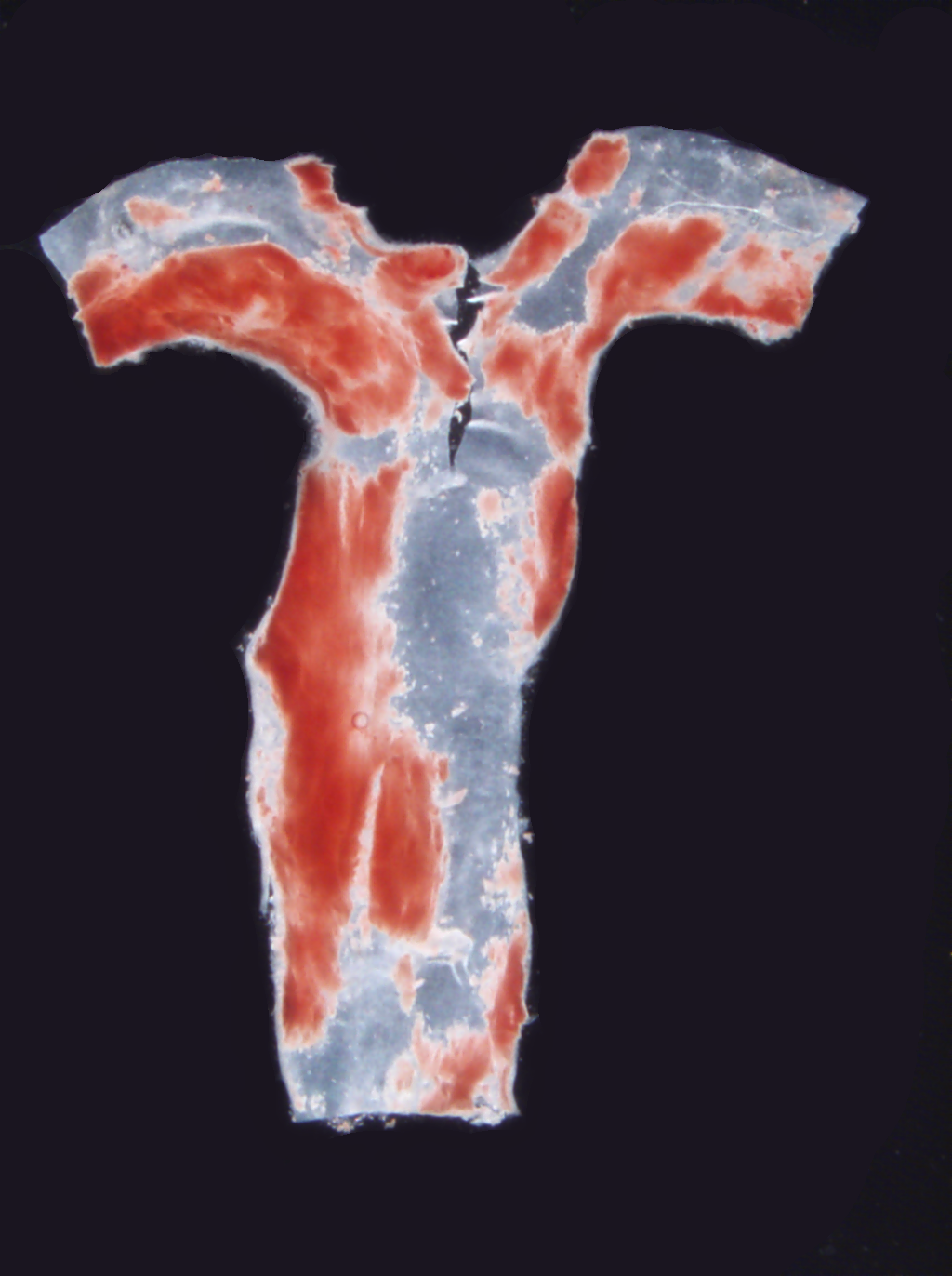


07


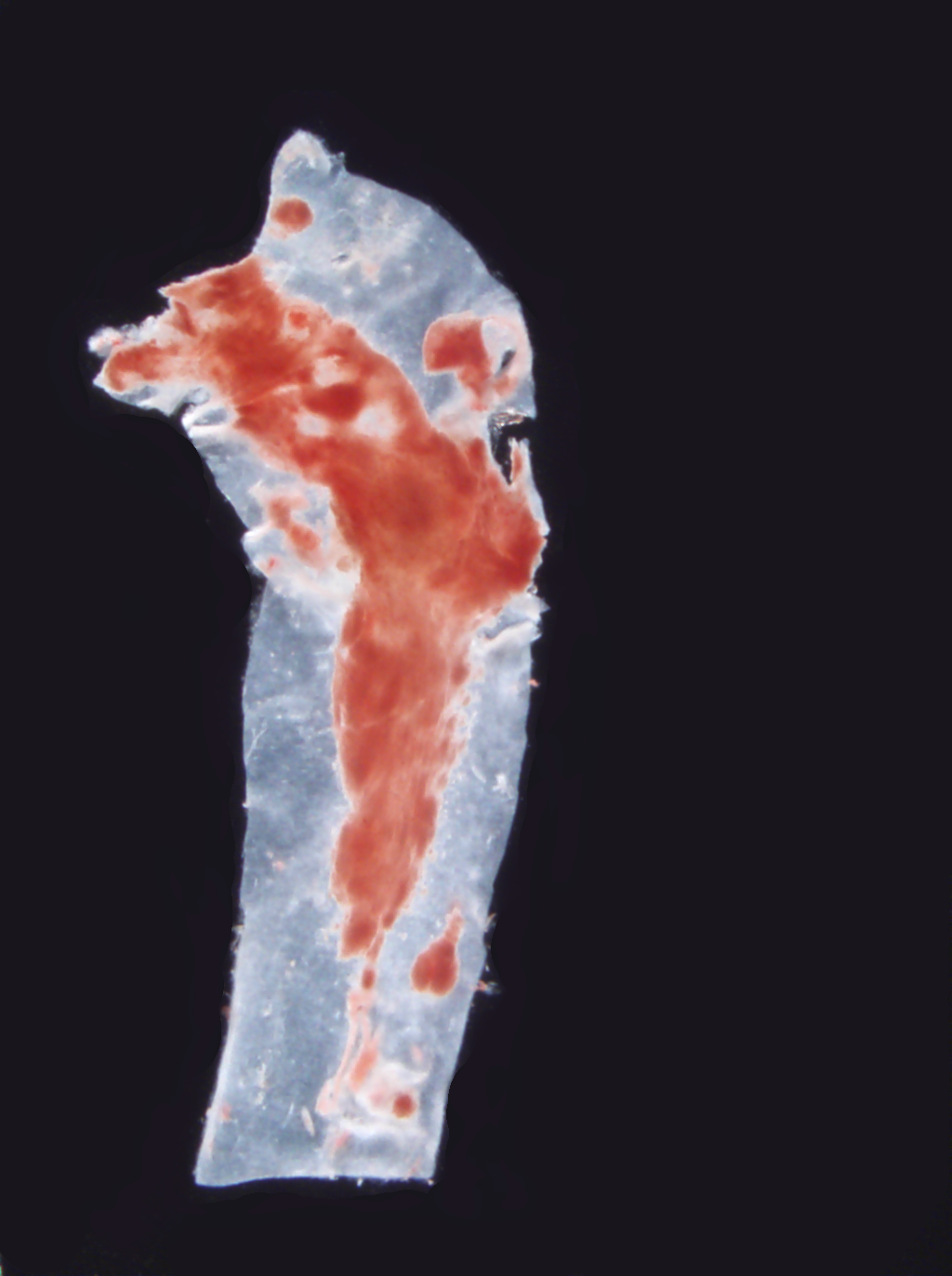


17


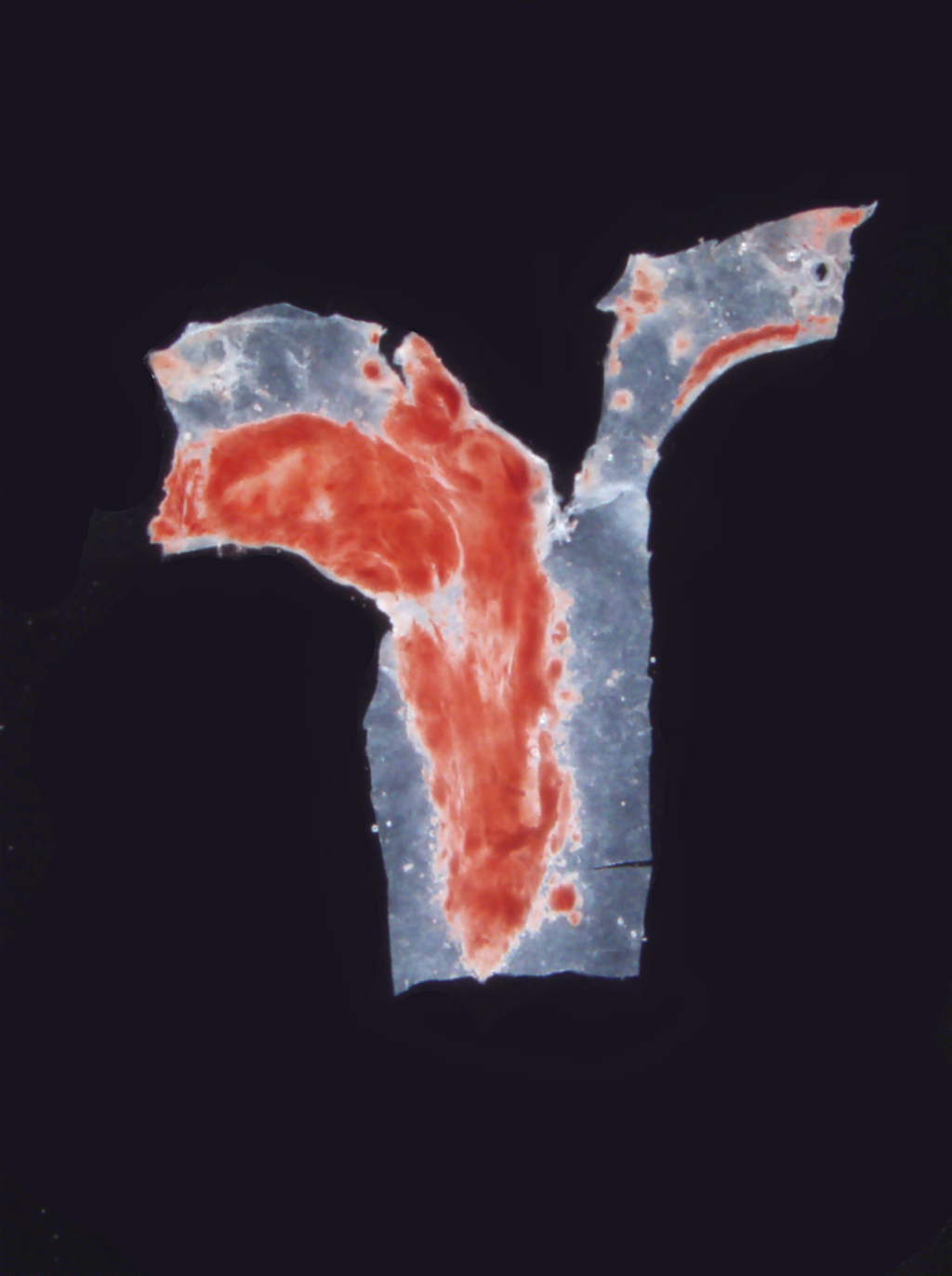


18

**2 mm**

Supplement: Figure S4 — En face assessment of atherosclerotic plaque in Ldlr-1KO-AD mice. Aortas from Ldlr-1KO fed atherogenic diet used for microCT analysis were dissected from the carcass and assessed by en face methods and Sudan IV staining to allow comparison of the aortic arch plaque using the 2 techniques. Lesional surface areas are reported in Table 1. Although the entire descending aorta was analyzed and reported, here we show only the aortic arch. (DOC) [file pone.0018800.s004.doc]
