# Supplementary material for: A Volumetric Method for Quantifying Atherosclerosis in Mice by Using MicroCT: Comparison to En Face
Source: PLoS One. 2011 Apr 18;6(4):e18800. doi: 10.1371/journal.pone.0018800 (PMC3078927; doi:10.1371/journal.pone.0018800)
Supplement: Figure S3 — Comparison of microCT to en face . (A) Fold differences between the total intimal plaque surface area in Ldlr-2KO and -3KO aortas were calculated for each method. (B) The fold difference in total plaque volume determined by microCT was calculated between the two strains of mice. Region of the total aorta is described in Methods section. (DOC) [file pone.0018800.s003.doc]

**Figure S3**

**A**

**B**
