# Supplementary material for: A Volumetric Method for Quantifying Atherosclerosis in Mice by Using MicroCT: Comparison to En Face
Source: PLoS One. 2011 Apr 18;6(4):e18800. doi: 10.1371/journal.pone.0018800 (PMC3078927; doi:10.1371/journal.pone.0018800)
Supplement: Table S1 — Body weight and plasma lipid characteristic of Ldlr-2KO and -3KO mice (N = 4). Ldlr-3KO mice were obese, extremely hypercholesterolemic (including elevated HDL-cholesterol) and hypertriglyceridemic compared with Ldlr-2KO mice. The Ldlr-2KO mice were hypercholesterolemic compared with typical levels in wildtype mice. (DOC) [file pone.0018800.s005.doc]

**Table S**1.

| Genotype | Body Weight  (g) | Cholesterol  (mg/dL) | Triglycerides  (mg/dL) | HDL-cholesterol  (mg/dL) | NEFA  (mEq) |
| --- | --- | --- | --- | --- | --- |
| Ldlr-3KO | 64.8±1 | 2135.2±111 | 764.2±227 | 112.8±4 | 1.21±0.1 |
| Ldlr-2KO | 32.7±1 | 485.9±16 | 224.4±17 | 69.7±2 | 1.35±0.1 |
